# Supplementary material for: Randomized Crossover Trial Evaluating Detoxification of Tobacco Carcinogens by Broccoli Seed and Sprout Extract in Current Smokers
Source: Cancers (Basel). 2022 Apr 24;14(9):2129. doi: 10.3390/cancers14092129 (PMC9105060; doi:10.3390/cancers14092129)
Supplement: Supplementary file 1 [file cancers-14-02129-s001.zip › cancers-1662635-supplementary.pdf]

cancers

MDPI

Supplementary Materials

Randomized Crossover Trial Evaluating Detoxification of Tobacco Carcinogens by Broccoli Seed and Sprout Extract in Current Smokers

Julie E. Bauman, Chiu-Hsieh Hsu, Sara Centuori, Jose Guillen-Rodriguez, Linda L. Garland, Emily Ho, Megha Padi, Vignesh Bageerathan, Lisa Bengtson, Malgorzata Wojtowicz, Eva Szabo and H.-H. Sherry Chow

Table S1. Correlation Between Change in Gene Expression and Effective SFN Dose.

Correlation Value	Gene
0.38	NQO1
0.34	GCLM
0.27	APLNR
0.26	SLC7A11
0.25	IL34
0.24	PARP12
0.23	BAX
0.23	BRD4
0.22	BBC3
0.21	GCLC
0.21	DDB2
0.20	CASP3
0.20	ARID1A
0.19	SLAMF7
0.19	CD2
0.18	HRAS
0.18	WNT5A
0.17	ARG2
0.17	CCNB1
0.17	IFI16
0.16	VEGFB
0.16	WNT2B
0.16	LGALS9
0.16	CSF2
0.16	ENO1
0.16	PSMB5
0.15	IKBKG
0.15	TRIM21
0.15	FADD
0.15	GAS1
0.14	MS4A2
0.14	SIGLEC1
0.14	P4HA2
0.14	ROBO4
0.14	MAML2
0.14	TNFRSF10D

0.13	NRAS
0.13	GNLY
0.13	ERBB2
0.13	NT5E
0.13	PLOD2
0.13	SGK1
0.13	EIF2AK2
0.12	NFE2L2
0.12	HERC6
0.12	TRAF1
0.12	GHR
0.12	HMOX1
0.12	HDAC5
0.11	FAP
0.11	NOTCH2
0.11	PRKACB
0.11	Mif
0.11	IL22RA1
0.11	TP53
0.11	MTOR
0.11	EPM2AIP1
0.10	SLC7A5
0.10	BAMBI
0.10	OTOA
0.10	PGPEP1
0.10	ZC3H12A
0.10	SOX2
0.10	FPR3
0.10	RASAL1
0.10	GZMM
0.10	CD247
0.10	BRCA2
0.10	SERPINH1
0.10	MS4A1
0.10	ATF3
0.09	ANGPT2
0.09	BRD3
0.09	GLI1
0.09	CXorf36
0.09	CDC25C
0.09	POLD1
0.09	PMS2
0.09	MX1
0.09	CRABP2
0.09	CD3G
0.09	COL6A3
0.08	VEGFC
0.08	VTCN1
0.08	COL11A2
0.08	WNT4
0.08	IRF3
0.08	TAPBPL
0.08	EPCAM

0.08	LAMA1
0.08	HMGB1
0.08	IFI27
0.08	HSPB1
0.08	TNFSF18
0.08	KAT2B
0.08	RIPK1
0.08	CCL18
0.07	IRF8
0.07	CD86
0.07	GLUL
0.07	PPARG
0.07	TLR5
0.07	CASP9
0.07	MICA
0.07	TWF1
0.07	S100A8
0.07	DEPTOR
0.07	BLK
0.07	CEP55
0.07	CXCL13
0.07	INHBA
0.06	ADORA2A
0.06	BIRC3
0.06	TIGIT
0.06	MXI1
0.06	Defb1
0.06	MAGEA12
0.06	CDC20
0.06	BNIP3L
0.06	PRLR
0.06	C7
0.06	EIF2B4
0.06	IKBKB
0.06	H2AFX
0.06	EGFR
0.06	CD84
0.06	ITGA2
0.06	ITGB8
0.06	EOMES
0.06	PDCD1
0.06	CXCL10
0.06	ALDOC
0.06	SMAP1
0.06	ICOS
0.05	SOX10
0.05	AKR1C1
0.05	PRR5
0.05	CSF1
0.05	GLUD1
0.05	VEGFA
0.05	HLA.DOA
0.05	OLR1

0.05	COL11A1
0.05	LILRB4
0.05	CCND1
0.05	SPP1
0.05	DTX4
0.05	LY9
0.05	IL11
0.05	WNT7B
0.04	KIF2C
0.04	LRRC32
0.04	MET
0.04	IFNAR1
0.04	FZD8
0.04	HDC
0.04	CCL7
0.04	PIAS4
0.04	MLH1
0.04	CCL4
0.04	CMKLR1
0.04	TCF3
0.04	MAPK10
0.04	CSF3
0.04	GOT1
0.04	MAGEA1
0.04	MS4A4A
0.04	TNFRSF11A
0.04	MICB
0.04	HEY1
0.04	SERPINA1
0.04	IL21R
0.04	LIF
0.04	FGF18
0.04	CTNNB1
0.04	IL17A
0.03	PRKAA2
0.03	SIRPA
0.03	IL1RN
0.03	CXCR6
0.03	IFIT1
0.03	PTPN11
0.03	Traf6
0.03	KRAS
0.03	LOXL2
0.03	CXCL5
0.03	TTC30A
0.03	CD70
0.03	MMP7
0.03	DLL4
0.03	SRP54
0.03	SMAD5
0.03	GNG4
0.03	CD3E
0.03	BATF3

0.03	GZMK
0.03	TNFSF4
0.03	LAG3
0.03	IL2
0.03	UBE2C
0.03	LAMC2
0.03	CDKN1C
0.02	HMGA1
0.02	DTX3L
0.02	CCR4
0.02	CX3CL1
0.02	CD40
0.02	TAP2
0.02	HLA.DQA1
0.02	KLRK1
0.02	CDK6
0.02	DUSP1
0.02	PVRIG
0.02	BAD
0.02	PLA2G2A
0.02	CXCL14
0.02	SPIB
0.02	FLNB
0.02	IDO1
0.02	COMP
0.02	CD8B
0.02	APC
0.01	PIK3CA
0.01	RPTOR
0.01	WNT11
0.01	RB1
0.01	BRCA1
0.01	ICAM5
0.01	ID4
0.01	MYD88
0.01	TSLP
0.01	RBL2
0.01	ARG1
0.01	RRM2
0.01	VCAM1
0.01	SLC2A1
0.01	HK2
0.01	PTCD2
0.01	NOD2
0.01	NBN
0.01	BIRC5
0.01	KIT
0.01	ZEB2
0.01	IFI6
0.01	DKK1
0.01	CD28
0.01	TNFRSF8
0.01	PDGFRB

0.01	LYZ
0.01	ZEB1
0.01	MSH6
0.01	HSPA4
0.01	TNFSF8
0.01	BRIP1
0.00	CDKN2A
0.00	IL33
0.00	Ppia
0.00	S100A9
0.00	ELOB
0.00	RUNX3
0.00	PKM
0.00	EZH2
0.00	IL18
0.00	CD1C
0.00	FGF9
0.00	PDGFB
0.00	GPR160
0.00	HDAC11
0.00	CD6
0.00	CDKN1A
0.00	MSH2
0.00	XCL1.2
0.00	VCAN
0.00	TNFSF10
0.00	IL24
−0.01	IL11RA
−0.01	CTSW
−0.01	CCR5
−0.01	SAMSN1
−0.01	Lyve1
−0.01	CCL13
−0.01	HDAC3
−0.01	IL2RB
−0.01	CD209
−0.01	OAS1
−0.01	PTEN
−0.01	SOCS1
−0.01	CSF2RB
−0.01	TGFBR1
−0.01	E2F3
−0.01	MMRN2
−0.01	TNFRSF11B
−0.01	MLANA
−0.01	NFIL3
−0.01	C1QB
−0.01	CD274
−0.01	BLM
−0.01	MAP3K5
−0.02	CCL2
−0.02	COL5A1
−0.02	MAP3K12

−0.02	PDK1
−0.02	NKG7
−0.02	RORC
−0.02	RPS6KB1
−0.02	BNIP3
−0.02	RPL7A
−0.02	ROR2
−0.02	CCNA1
−0.02	ESR1
−0.02	ADM
−0.02	AKR1B10
−0.02	SFRP4
−0.02	CLECL1
−0.02	SPRY4
−0.02	CCNE1
−0.02	API5
−0.02	TRAT1
−0.02	OAS2
−0.02	WNT2
−0.02	MS4A6A
−0.02	ACVR1C
−0.02	ZAP70
−0.02	PC
−0.02	CXCL12
−0.02	FSTL3
−0.02	CD58
−0.03	RNLS
−0.03	CCL8
−0.03	IL18R1
−0.03	JAG1
−0.03	PRKX
−0.03	PIK3CD
−0.03	MRC1
−0.03	CD45RB
−0.03	CYBB
−0.03	AQP9
−0.03	DEFB134
−0.03	TDO2
−0.03	SIRPB2
−0.03	CD48
−0.03	PSMB8
−0.03	LAMB3
−0.03	BMP2
−0.03	PRF1
−0.03	STAT2
−0.03	TLR8
−0.03	S100A12
−0.03	CDKN2B
−0.03	MYCT1
−0.03	NFATC2
−0.03	IGF2R
−0.03	ALDOA
−0.04	ARNT2

−0.04	IFI35
−0.04	RAD50
−0.04	CNTFR
−0.04	SAMD9
−0.04	TYMS
−0.04	SELE
−0.04	TLR9
−0.04	PF4
−0.04	DUSP5
−0.04	PFKM
−0.04	CXCL9
−0.04	SFXN1
−0.04	IRF9
−0.04	DAB2
−0.04	SNCA
−0.04	FLT1
−0.04	SYK
−0.04	CCL3.L1
−0.04	APH1B
−0.04	APOL6
−0.04	EIF5AL1
−0.04	ADGRE1
−0.04	CCND3
−0.04	ITGA6
−0.04	LDHA
−0.04	ITGAE
−0.04	TNFRSF9
−0.04	ERO1A
−0.04	TNFRSF4
−0.04	CASP8
−0.04	VSIR
−0.05	JAK2
−0.05	FASLG
−0.05	RSAD2
−0.05	THBS1
−0.05	CDH1
−0.05	TNFAIP6
−0.05	ULBP2
−0.05	MYC
−0.05	PFKFB3
−0.05	MB21D1
−0.05	ITGAV
−0.05	COL17A1
−0.05	C5
−0.05	DNMT1
−0.05	RASGRF1
−0.05	PVR
−0.05	CD163
−0.05	ITGB3
−0.05	IL2RA
−0.05	RICTOR
−0.05	STAT4
−0.05	CD3D

−0.05	FYN
−0.05	CCND2
−0.05	TLR3
−0.05	HELLS
−0.05	HNF1A
−0.05	KIR2DL3
−0.05	CD69
−0.05	CTAG1B
−0.06	SERPINB5
−0.06	CXCL11
−0.06	CD276
−0.06	FCGR2B
−0.06	IL6
−0.06	REN
−0.06	IRF1
−0.06	CD36
−0.06	FAM124B
−0.06	P4HA1
−0.06	EXO1
−0.06	CD8A
−0.06	BID
−0.06	GOT2
−0.06	THY1
−0.06	KLRD1
−0.06	IL10
−0.06	TPI1
−0.06	KLRB1
−0.06	HAVCR2
−0.06	MFGE8
−0.06	TLR2
−0.06	IFNA1
−0.06	WDR76
−0.06	CCL5
−0.06	MMP1
−0.06	KIR3DL1
−0.06	IL12RB2
−0.07	SREBF1
−0.07	CD79B
−0.07	TNFRSF18
−0.07	EDN1
−0.07	CD38
−0.07	PIK3CG
−0.07	MELK
−0.07	CDK2
−0.07	ICAM2
−0.07	TYMP
−0.07	ANGPT1
−0.07	CD19
−0.07	TLR4
−0.07	NID2
−0.07	JAK1
−0.07	CLEC5A
−0.07	CD45RA

−0.07	CDH5
−0.07	LY96
−0.07	CDH11
−0.07	HLA.DPA1
−0.07	TICAM1
−0.07	TGFB2
−0.07	WNT3A
−0.07	CDH2
−0.07	RELA
−0.08	CHUK
−0.08	IRF5
−0.08	FCAR
−0.08	HES1
−0.08	NFKB2
−0.08	RAD51
−0.08	FUT4
−0.08	DPP4
−0.08	CD4
−0.08	FGF13
−0.08	AKT1
−0.08	NF1
−0.08	TWIST2
−0.08	MAGEA4
−0.08	GZMA
−0.08	IFNGR1
−0.08	FCGR2A
−0.08	NOS2
−0.08	CCNO
−0.08	PIK3R1
−0.08	BCAT1
−0.08	NDUFA4L2
−0.08	FANCA
−0.08	VHL
−0.08	ITPK1
−0.08	IL7R
−0.08	CLEC14A
−0.08	TNFSF12
−0.09	IFIT2
−0.09	TNFRSF10C
−0.09	HLA.A
−0.09	TGFBR2
−0.09	LAIR1
−0.09	SLC11A1
−0.09	CLEC4E
−0.09	TPSAB1.B2
−0.09	NFAM1
−0.09	TNFRSF1A
−0.09	ICOSLG
−0.09	BBS1
−0.09	MKI67
−0.09	PRKCA
−0.09	ANLN
−0.09	CCL19

−0.09	AXIN1
−0.09	SELP
−0.09	EIF4EBP1
−0.10	HLA.DQB1
−0.10	CXCL6
−0.10	GZMH
−0.10	IFIH1
−0.10	IFIT3
−0.10	FZD9
−0.10	IL16
−0.10	PTPRC
−0.10	MAP3K7
−0.10	TLR1
−0.10	GZMB
−0.10	CD47
−0.10	CAT
−0.10	TGFB1
−0.10	PIK3R2
−0.10	EGF
−0.10	ITGA4
−0.10	BTLA
−0.10	PIK3R5
−0.10	KIR3DL2
−0.10	HLA.DPB1
−0.10	IER3
−0.10	LTBP1
−0.11	P2RY13
−0.11	SELL
−0.11	ADAM12
−0.11	WNT5B
−0.11	IFNG
−0.11	SLC16A1
−0.11	OLFML2B
−0.11	TMEM140
−0.11	TNFRSF10B
−0.11	MAGEA3.A6
−0.11	CLEC7A
−0.11	GPSM3
−0.11	HLA.DMB
−0.11	CCL14
−0.11	IL6R
−0.11	NLRP3
−0.12	F2RL1
−0.12	NCAM1
−0.12	ISG15
−0.12	PDCD1LG2
−0.12	IFNGR2
−0.12	B2M
−0.12	HLA.B
−0.12	TNFSF13
−0.12	FBP1
−0.12	TPM1
−0.12	ATM

−0.12	IL1B
−0.12	LILRB2
−0.12	TWIST1
−0.12	FPR1
−0.12	LILRA5
−0.12	ITGB2
−0.13	IRF2
−0.13	NFKBIE
−0.13	NECTIN1
−0.13	APOE
−0.13	GBP2
−0.13	BCL6B
−0.13	CCR2
−0.13	PALMD
−0.13	CES3
−0.13	CCL21
−0.13	SMPD1
−0.13	CD40LG
−0.13	EGR1
−0.13	PSMB10
−0.13	CD44
−0.13	LCK
−0.13	MRE11
−0.13	CTLA4
−0.13	CXCR2
−0.13	RPL23
−0.13	TAP1
−0.13	AREG
−0.13	CASP1
−0.13	FCGRT
−0.13	CSF3R
−0.13	FAS
−0.14	GLS
−0.14	NGFR
−0.14	TGFB3
−0.14	CPA3
−0.14	UBE2T
−0.14	HLA.DOB
−0.14	CXCR3
−0.14	CENPF
−0.14	PCK2
−0.14	STAT3
−0.14	KDR
−0.14	LILRA1
−0.14	DUSP2
−0.15	GMIP
−0.15	HCK
−0.15	GIMAP6
−0.15	PARP4
−0.15	IL15
−0.15	ROCK1
−0.15	HIF1A
−0.15	MAGEB2

−0.15	TNFRSF17
−0.15	CD5
−0.15	TNFSF9
−0.15	MAGEC2
−0.15	CD14
−0.15	CX3CR1
−0.15	HK1
−0.15	SH2D1A
−0.15	STAT1
−0.16	AXL
−0.16	MAP3K8
−0.16	LDHB
−0.16	TLR7
−0.16	TAF3
−0.16	RIPK3
−0.16	TNKS
−0.16	FCRL2
−0.16	MAGEC1
−0.16	SBNO2
−0.16	PTGS2
−0.16	CXCL8
−0.16	SFRP1
−0.16	ITGAM
−0.16	SIGLEC5
−0.17	MGMT
−0.17	STC1
−0.17	NEIL1
−0.17	CD68
−0.17	SOD2
−0.17	CD300A
−0.17	PECAM1
−0.17	PROM1
−0.17	MARCO
−0.17	CST2
−0.17	IRF7
−0.17	SIGLEC8
−0.17	CD96
−0.17	HLA.DRB5
−0.17	IL4
−0.17	PDZK1IP1
−0.17	OAS3
−0.17	NLRC5
−0.17	ITGAL
−0.18	HLA.F
−0.18	FOXP3
−0.18	IL10RA
−0.18	CTSS
−0.18	PPARGC1B
−0.18	CXCL1
−0.18	OASL
−0.18	BCL2L1
−0.18	FCGR3A.B
−0.18	LILRA3

−0.18	A2M
−0.18	GBP1
−0.18	IHH
−0.18	TREM2
−0.19	COL4A5
−0.19	NECTIN2
−0.19	GPC4
−0.19	HLA.DRA
−0.19	TBX21
−0.19	TREM1
−0.19	HLA.E
−0.19	TNFSF13B
−0.19	IFITM2
−0.19	PDGFA
−0.19	HLA.DMA
−0.19	CBLC
−0.19	NFKB1
−0.19	FOSL1
−0.19	CEBPB
−0.20	CXCL16
−0.20	PNOC
−0.20	IFITM1
−0.20	PLA1A
−0.20	CD45RO
−0.20	SHC2
−0.20	CD7
−0.20	SNAI1
−0.20	GIMAP4
−0.20	Ceacam1
−0.20	CEACAM3
−0.21	WNT10A
−0.21	IL32
−0.21	TNFRSF25
−0.21	DLL1
−0.21	ICAM1
−0.21	C2
−0.21	ITGAX
−0.21	GBP4
−0.21	MFNG
−0.21	NOTCH1
−0.22	CD80
−0.22	IL1A
−0.22	UBA7
−0.22	MMP9
−0.22	IL2RG
−0.22	TNFRSF14
−0.22	CD27
−0.22	CCL22
−0.23	SLC1A5
−0.23	JAG2
−0.23	CD244
−0.23	SOX11
−0.23	Fcer2a

−0.23	TBXAS1
−0.23	THBD
−0.23	RELN
−0.23	CD79A
−0.23	PARP9
−0.24	PTGER4
−0.24	ENTPD1
−0.24	NFKBIA
−0.24	HLA.DQA2
−0.24	FCGR1A
−0.24	TCL1A
−0.24	IL1R2
−0.24	ICAM3
−0.25	C1QA
−0.25	CXCR4
−0.25	TAPBP
−0.25	TMEM173
−0.26	FAM30A
−0.26	ANGPTL4
−0.26	FCN1
−0.26	NCR1
−0.27	BCL2
−0.27	IRF4
−0.27	HSD11B1
−0.27	FGFR1
−0.29	CD74
−0.29	CSF1R
−0.30	JAK3
−0.30	TNFRSF1B
−0.30	RAD51C
−0.30	CXCL3
−0.31	HLA.C
−0.31	CXCL2
−0.31	RELB
−0.32	PSMB9
−0.32	RIPK2
−0.32	TNF
−0.34	HLA.DRB1
−0.34	HDAC4
−0.34	ITGA1
−0.34	LTB
−0.34	CCL20
−0.35	C5AR1
−0.36	TIE1
−0.42	TNFAIP3

Table S2. Gene Ontology Over-Representation Analysis of 30 Top Correlated Genes.

ID	Description	Gene Ratio	Bg Ratio	*p*-value	Adjusted *p*-value	q value	Gene ID
GO:0006520	cellular amino acid metabolic process	6/30	21/751	8.65×10^{-5}	0.07435	0.070071	NQO1/GCLM/SLC7A11/GCLC/ARG2/PSMB5
GO:1901564	organonitrogen compound metabolic process	26/30	400/751	9.16×10^{-5}	0.07435	0.070071	NQO1/GCLM/APLNR/SLC7A11/IL34/PARP12/BAX/BRD4/BBC3/GCLC/DDB2/CASP3/HRAS/WNT5A/ARG2/CCNB1/IFI16/VEGFB/LGALS9/CSF2/ENO1/PSMB5/IKBKG/TRIM21/FADD/GAS1
GO:0033554	cellular response to stress	17/30	194/751	0.000245	0.119947	0.113044	NQO1/SLC7A11/BAX/BRD4/BBC3/DDB2/CASP3/HRAS/WNT5A/ARG2/CCNB1/IFI16/WNT2B/LGALS9/ENO1/PSMB5/IKBKG
GO:0071887	leukocyte apoptotic process	7/30	38/751	0.000418	0.119947	0.113044	SLC7A11/BAX/CASP3/WNT5A/ARG2/LGALS9/FADD
GO:0009064	glutamine family amino acid metabolic process	4/30	11/751	0.000564	0.119947	0.113044	GCLM/SLC7A11/GCLC/ARG2
GO:0006508	proteolysis	13/30	132/751	0.000696	0.119947	0.113044	BAX/BBC3/DDB2/CASP3/CCNB1/IFI16/LGALS9/ENO1/PSMB5/IKBKG/TRIM21/FADD/GAS1
GO:2000106	regulation of leukocyte apoptotic process	6/30	30/751	0.000734	0.119947	0.113044	SLC7A11/BAX/WNT5A/ARG2/LGALS9/FADD
GO:0016485	protein processing	5/30	20/751	0.000735	0.119947	0.113044	CASP3/IFI16/ENO1/FADD/GAS1
GO:0070231	T cell apoptotic process	5/30	20/751	0.000735	0.119947	0.113044	BAX/WNT5A/ARG2/LGALS9/FADD
GO:0070997	neuron death	9/30	68/751	0.000739	0.119947	0.113044	NQO1/GCLM/SLC7A11/BAX/CASP3/HRAS/WNT5A/IKBKG/FADD
GO:0043029	T cell homeostasis	4/30	12/751	0.000823	0.121528	0.114533	BAX/CASP3/LGALS9/FADD
GO:0070228	regulation of lymphocyte apoptotic process	5/30	21/751	0.000937	0.126855	0.119554	BAX/WNT5A/ARG2/LGALS9/FADD
GO:0001776	leukocyte homeostasis	5/30	23/751	0.001464	0.15969	0.150499	SLC7A11/BAX/CASP3/LGALS9/FADD
GO:0051604	protein maturation	5/30	23/751	0.001464	0.15969	0.150499	CASP3/IFI16/ENO1/FADD/GAS1

GO:0070232	regulation of T cell apoptotic process	4/30	14/751	0.001573	0.15969	0.150499	WNT5A/ARG2/LGALS9/FADD
GO:1901605	alpha-amino acid metabolic process	4/30	14/751	0.001573	0.15969	0.150499	GCLM/SLC7A11/GCLC/ARG2
GO:0009628	response to abiotic stimulus	13/30	144/751	0.001683	0.160744	0.151492	NQO1/SLC7A11/BAX/GCLC/DDB2/CASP3/HRAS/CCNB1/IFI16/VEGFB/ENO1/PSMB5/FADD
GO:1901214	regulation of neuron death	8/30	62/751	0.001876	0.169273	0.15953	NQO1/GCLM/SLC7A11/BAX/CASP3/HRAS/WNT5A/IKBKG
GO:0051247	positive regulation of protein metabolic process	16/30	211/751	0.002583	0.213411	0.201127	APLNR/IL34/BAX/BRD4/BBC3/CASP3/HRAS/WNT5A/CCNB1/IFI16/VEGFB/LGALS9/CSF2/ENO1/IKBKG/FADD
GO:0070227	lymphocyte apoptotic process	5/30	26/751	0.002628	0.213411	0.201127	BAX/WNT5A/ARG2/LGALS9/FADD
GO:0006915	apoptotic process	18/30	260/751	0.003317	0.253738	0.239133	NQO1/GCLM/SLC7A11/BAX/BBC3/GCLC/CASP3/CD2/HRAS/WNT5A/ARG2/IFI16/LGALS9/CSF2/ENO1/IKBKG/FADD/GAS1
GO:0002260	lymphocyte homeostasis	4/30	17/751	0.003437	0.253738	0.239133	BAX/CASP3/LGALS9/FADD
GO:0034641	cellular nitrogen compound metabolic process	21/30	337/751	0.004119	0.272821	0.257118	NQO1/GCLM/SLC7A11/BAX/BRD4/GCLC/DDB2/CASP3/ARID1A/HRAS/WNT5A/ARG2/CCNB1/IFI16/LGALS9/CSF2/ENO1/PSMB5/IKBKG/TRIM21/FADD
GO:0032268	regulation of cellular protein metabolic process	18/30	265/751	0.00423	0.272821	0.257118	APLNR/IL34/BAX/BRD4/BBC3/CASP3/HRAS/WNT5A/CCNB1/IFI16/VEGFB/LGALS9/CSF2/ENO1/IKBKG/TRIM21/FADD/GAS1
GO:0044267	cellular protein metabolic process	21/30	339/751	0.0045	0.272821	0.257118	APLNR/IL34/PARP12/BAX/BRD4/BBC3/DDB2/CASP3/HRAS/WNT5A/CCNB1/IFI16/VEGFB/LGALS9/CSF2/ENO1/PSMB5/IKBKG/TRIM21/FADD/GAS1
GO:0031323	regulation of cellular metabolic process	24/30	421/751	0.004805	0.272821	0.257118	NQO1/APLNR/SLC7A11/IL34/BAX/BRD4/BBC3/GCLC/CASP3/ARID1A/HRAS/WNT5A/ARG2/CCNB1/IFI16/VEGFB/LGALS9/CSF2/ENO1/PSMB5/IKBKG/TRIM21/FADD/GAS1
GO:0010638	positive regulation of organelle organization	7/30	57/751	0.005131	0.272821	0.257118	BAX/BRD4/BBC3/HRAS/WNT5A/CCNB1/CSF2
GO:0060548	negative regulation of cell death	13/30	162/751	0.005232	0.272821	0.257118	NQO1/GCLM/SLC7A11/BAX/GCLC/CASP3/HRAS/WNT5A/ARG2/CSF2/ENO1/IKBKG/FADD
GO:0071478	cellular response to radiation	4/30	19/751	0.005291	0.272821	0.257118	BAX/DDB2/HRAS/IFI16

GO:0012501	programmed cell death	18/30	270/751	0.005352	0.272821	0.257118	NQO1/GCLM/SLC7A11/BAX/BBC3/GCLC/CASP3/CD2/HRAS/WNT5A/ARG2/IFI16/LGALS9/CSF2/ENO1/IKBKG/FADD/GAS1
GO:0045088	regulation of innate immune response	8/30	73/751	0.005482	0.272821	0.257118	HRAS/WNT5A/IFI16/LGALS9/PSMB5/IKBKG/TRIM21/FADD
GO:0044271	cellular nitrogen compound biosynthetic process	19/30	295/751	0.005703	0.272821	0.257118	NQO1/GCLM/SLC7A11/BAX/BRD4/GCLC/ARID1A/HRAS/WNT5A/ARG2/CCNB1/IFI16/LGALS9/CSF2/ENO1/PSMB5/IKBKG/TRIM21/FADD
GO:0002088	lens development in camera-type eye	3/30	10/751	0.005721	0.272821	0.257118	SLC7A11/WNT5A/WNT2B
GO:0006790	sulfur compound metabolic process	3/30	10/751	0.005721	0.272821	0.257118	GCLM/SLC7A11/GCLC
GO:0032729	positive regulation of interferon-gamma production	5/30	31/751	0.005888	0.272821	0.257118	CD2/HRAS/WNT5A/LGALS9/FADD
GO:0032270	positive regulation of cellular protein metabolic process	15/30	206/751	0.006111	0.272821	0.257118	APLNR/IL34/BAX/BRD4/BBC3/HRAS/WNT5A/CCNB1/IFI16/VEGFB/LGALS9/CSF2/ENO1/IKBKG/FADD
GO:0002831	regulation of response to biotic stimulus	9/30	91/751	0.006236	0.272821	0.257118	HRAS/WNT5A/ARG2/IFI16/LGALS9/PSMB5/IKBKG/TRIM21/FADD
GO:0002832	negative regulation of response to biotic stimulus	4/30	20/751	0.00643	0.272821	0.257118	ARG2/IFI16/LGALS9/TRIM21
GO:0051402	neuron apoptotic process	6/30	45/751	0.006572	0.272821	0.257118	NQO1/GCLM/BAX/CASP3/HRAS/FADD
GO:0051246	regulation of protein metabolic process	18/30	275/751	0.00672	0.272821	0.257118	APLNR/IL34/BAX/BRD4/BBC3/CASP3/HRAS/WNT5A/CCNB1/IFI16/VEGFB/LGALS9/CSF2/ENO1/IKBKG/TRIM21/FADD/GAS1
GO:0060341	regulation of cellular localization	8/30	76/751	0.007068	0.279942	0.263829	APLNR/SLC7A11/BAX/BBC3/HRAS/WNT5A/LGALS9/GAS1
GO:0090305	nucleic acid phosphodiester bond hydrolysis	3/30	11/751	0.007654	0.280355	0.264218	BAX/DDB2/CASP3
GO:0038034	signal transduction in absence of ligand	4/30	21/751	0.007723	0.280355	0.264218	BAX/CASP3/CSF2/FADD
GO:0097192	extrinsic apoptotic signaling pathway in absence of ligand	4/30	21/751	0.007723	0.280355	0.264218	BAX/CASP3/CSF2/FADD
GO:2001056	positive regulation of cysteine-type endopeptidase activity	5/30	33/751	0.007768	0.280355	0.264218	BAX/BBC3/IFI16/LGALS9/FADD

GO:0008219	cell death	18/30	281/751	0.008746	0.288107	0.271524	NQO1/GCLM/SLC7A11/BAX/BBC3/GCLC/CASP3/CD2/HRAS/WNT5A/ARG2/IFI16/LGALS9/CSF2/ENO1/IKBKG/FADD/GAS1
GO:0030162	regulation of proteolysis	8/30	79/751	0.008985	0.288107	0.271524	BAX/BBC3/IFI16/LGALS9/ENO1/TRIM21/FADD/GAS1
GO:0019752	carboxylic acid metabolic process	7/30	63/751	0.009054	0.288107	0.271524	NQO1/GCLM/SLC7A11/GCLC/ARG2/ENO1/PSMB5
GO:0043436	oxoacid metabolic process	7/30	63/751	0.009054	0.288107	0.271524	NQO1/GCLM/SLC7A11/GCLC/ARG2/ENO1/PSMB5
GO:0045089	positive regulation of innate immune response	6/30	48/751	0.009088	0.288107	0.271524	HRAS/WNT5A/IFI16/PSMB5/IKBKG/FADD
GO:0051171	regulation of nitrogen compound metabolic process	23/30	409/751	0.00919	0.288107	0.271524	NQO1/APLNR/SLC7A11/IL34/BAX/BRD4/BBC3/GCLC/CASP3/ARID1A/HRAS/WNT5A/CCNB1/IFI16/VEGFB/LGALS9/CSF2/ENO1/PSMB5/IKBKG/TRIM21/FADD/GAS1
GO:0006082	organic acid metabolic process	7/30	64/751	0.009881	0.288107	0.271524	NQO1/GCLM/SLC7A11/GCLC/ARG2/ENO1/PSMB5
GO:2000108	positive regulation of leukocyte apoptotic process	3/30	12/751	0.009931	0.288107	0.271524	BAX/WNT5A/LGALS9
GO:0030900	forebrain development	5/30	35/751	0.010037	0.288107	0.271524	SLC7A11/BAX/CASP3/WNT5A/WNT2B
GO:0009314	response to radiation	6/30	49/751	0.010065	0.288107	0.271524	SLC7A11/BAX/DDB2/CASP3/HRAS/IFI16
GO:0043122	regulation of I-kappaB kinase/NF-kappaB signaling	6/30	49/751	0.010065	0.288107	0.271524	BRD4/WNT5A/LGALS9/IKBKG/TRIM21/FADD
GO:0044249	cellular biosynthetic process	20/30	333/751	0.010112	0.288107	0.271524	NQO1/GCLM/SLC7A11/BAX/BRD4/GCLC/ARID1A/HRAS/WNT5A/ARG2/CCNB1/IFI16/VEGFB/LGALS9/CSF2/ENO1/PSMB5/IKBKG/TRIM21/FADD
GO:0080090	regulation of primary metabolic process	23/30	413/751	0.010744	0.28961	0.272941	NQO1/APLNR/SLC7A11/IL34/BAX/BRD4/BBC3/GCLC/CASP3/ARID1A/HRAS/WNT5A/CCNB1/IFI16/VEGFB/LGALS9/CSF2/ENO1/PSMB5/IKBKG/TRIM21/FADD/GAS1
GO:0021537	telencephalon development	4/30	23/751	0.010801	0.28961	0.272941	SLC7A11/BAX/CASP3/WNT5A
GO:0010950	positive regulation of endopeptidase activity	5/30	36/751	0.011328	0.28961	0.272941	BAX/BBC3/IFI16/LGALS9/FADD

GO:0010952	positive regulation of peptidase activity	5/30	36/751	0.011328	0.28961	0.272941	BAX/BBC3/IFI16/LGALS9/FADD
GO:1901215	negative regulation of neuron death	5/30	36/751	0.011328	0.28961	0.272941	GCLM/SLC7A11/BAX/HRAS/IKBKG
GO:0010941	regulation of cell death	16/30	241/751	0.011337	0.28961	0.272941	NQO1/GCLM/SLC7A11/BAX/BBC3/GCLC/CASP3/HRAS/WNT5A/ARG2/LGALS9/CSF2/ENO1/IKBKG/FADD/GAS1
GO:0042981	regulation of apoptotic process	15/30	219/751	0.011413	0.28961	0.272941	NQO1/GCLM/SLC7A11/BAX/BBC3/GCLC/CASP3/HRAS/WNT5A/ARG2/LGALS9/CSF2/ENO1/FADD/GAS1
GO:0019538	protein metabolic process	21/30	362/751	0.011671	0.291597	0.274813	APLNR/IL34/PARP12/BAX/BRD4/BBC3/DDB2/CASP3/HRAS/WNT5A/CCNB1/IFI16/VEGFB/LGALS9/CSF2/ENO1/PSMB5/IKBKG/TRIM21/FADD/GAS1
GO:0030224	monocyte differentiation	3/30	13/751	0.012564	0.304535	0.287007	IL34/IFI16/CSF2
GO:0044106	cellular amine metabolic process	3/30	13/751	0.012564	0.304535	0.287007	NQO1/SLC7A11/PSMB5
GO:0045862	positive regulation of proteolysis	6/30	52/751	0.013449	0.305956	0.288346	BAX/BBC3/IFI16/LGALS9/ENO1/FADD
GO:0071214	cellular response to abiotic stimulus	6/30	52/751	0.013449	0.305956	0.288346	BAX/DDB2/CASP3/HRAS/IFI16/FADD
GO:0097193	intrinsic apoptotic signaling pathway	6/30	52/751	0.013449	0.305956	0.288346	BAX/BBC3/CASP3/HRAS/IFI16/ENO1
GO:0104004	cellular response to environmental stimulus	6/30	52/751	0.013449	0.305956	0.288346	BAX/DDB2/CASP3/HRAS/IFI16/FADD
GO:0006950	response to stress	25/30	476/751	0.013565	0.305956	0.288346	NQO1/GCLM/SLC7A11/IL34/BAX/BRD4/BBC3/GCLC/DDB2/CASP3/SLAMF7/HRAS/WNT5A/ARG2/CCNB1/IFI16/VEGFB/WNT2B/LGALS9/CSF2/ENO1/PSMB5/IKBKG/TRIM21/FADD
GO:0010212	response to ionizing radiation	4/30	25/751	0.014585	0.324476	0.3058	BAX/CASP3/HRAS/IFI16
GO:0009058	biosynthetic process	20/30	343/751	0.014961	0.326509	0.307716	NQO1/GCLM/SLC7A11/BAX/BRD4/GCLC/ARID1A/HRAS/WNT5A/ARG2/CCNB1/IFI16/VEGFB/LGALS9/CSF2/ENO1/PSMB5/IKBKG/TRIM21/FADD
GO:0009308	amine metabolic process	3/30	14/751	0.015561	0.326509	0.307716	NQO1/SLC7A11/PSMB5
GO:0043279	response to alkaloid	3/30	14/751	0.015561	0.326509	0.307716	NQO1/CASP3/FADD

GO:0043525	positive regulation of neuron apoptotic process	3/30	14/751	0.015561	0.326509	0.307716	NQO1/BAX/CASP3
GO:0043123	positive regulation of I-kappaB kinase/NF-kappaB signaling	5/30	39/751	0.015883	0.326509	0.307716	BRD4/LGALS9/IKBKG/TRIM21/FADD
GO:0043523	regulation of neuron apoptotic process	5/30	39/751	0.015883	0.326509	0.307716	NQO1/GCLM/BAX/CASP3/HRAS
GO:0009636	response to toxic substance	4/30	26/751	0.016759	0.340217	0.320635	NQO1/SLC7A11/BAX/CCNB1
GO:0051726	regulation of cell cycle	9/30	106/751	0.017173	0.341816	0.322142	BAX/BRD4/CASP3/HRAS/WNT5A/CCNB1/PSMB5/TRIM21/GAS1
GO:0002833	positive regulation of response to biotic stimulus	6/30	55/751	0.017575	0.341816	0.322142	HRAS/WNT5A/IFI16/PSMB5/IKBKG/FADD
GO:0019222	regulation of metabolic process	25/30	483/751	0.017598	0.341816	0.322142	NQO1/APLNR/SLC7A11/IL34/BAX/BRD4/BBC3/GCLC/CASP3/ARID1A/CD2/HRAS/WNT5A/ARG2/CCNB1/IFI16/VEGFB/LGALS9/CSF2/ENO1/PSMB5/IKBKG/TRIM21/FADD/GAS1
GO:0043067	regulation of programmed cell death	15/30	229/751	0.01768	0.341816	0.322142	NQO1/GCLM/SLC7A11/BAX/BBC3/GCLC/CASP3/HRAS/WNT5A/ARG2/LGALS9/CSF2/ENO1/FADD/GAS1
GO:0097194	execution phase of apoptosis	3/30	15/751	0.01893	0.361225	0.340434	BAX/BBC3/CASP3
GO:0043010	camera-type eye development	4/30	27/751	0.019129	0.361225	0.340434	SLC7A11/BAX/WNT5A/WNT2B
GO:0033043	regulation of organelle organization	8/30	90/751	0.019549	0.364916	0.343912	BAX/BRD4/BBC3/HRAS/WNT5A/CCNB1/CSF2/ENO1
GO:0006974	cellular response to DNA damage stimulus	7/30	74/751	0.021517	0.368241	0.347045	BAX/BRD4/DDB2/CASP3/CCNB1/IFI16/IKBKG
GO:0001822	kidney development	5/30	42/751	0.021543	0.368241	0.347045	BAX/WNT5A/ARG2/WNT2B/FADD
GO:0072001	renal system development	5/30	42/751	0.021543	0.368241	0.347045	BAX/WNT5A/ARG2/WNT2B/FADD
GO:0008406	gonad development	4/30	28/751	0.021699	0.368241	0.347045	BAX/CASP3/WNT5A/WNT2B
GO:0016579	protein deubiquitination	4/30	28/751	0.021699	0.368241	0.347045	DDB2/PSMB5/IKBKG/TRIM21
GO:0045137	development of primary sexual characteristics	4/30	28/751	0.021699	0.368241	0.347045	BAX/CASP3/WNT5A/WNT2B

GO:0070646	protein modification by small protein removal	4/30	28/751	0.021699	0.368241	0.347045	DDB2/PSMB5/IKBKG/TRIM21
GO:1901216	positive regulation of neuron death	4/30	28/751	0.021699	0.368241	0.347045	NQO1/BAX/CASP3/WNT5A
GO:0006979	response to oxidative stress	6/30	58/751	0.022518	0.368241	0.347045	NQO1/GCLM/SLC7A11/GCLC/CASP3/PSMB5
GO:0046006	regulation of activated T cell proliferation	3/30	16/751	0.022675	0.368241	0.347045	CASP3/LGALS9/FADD
GO:0048593	camera-type eye morphogenesis	3/30	16/751	0.022675	0.368241	0.347045	BAX/WNT5A/WNT2B
GO:0048736	appendage development	3/30	16/751	0.022675	0.368241	0.347045	SLC7A11/BAX/WNT5A
GO:0060173	limb development	3/30	16/751	0.022675	0.368241	0.347045	SLC7A11/BAX/WNT5A
GO:0051173	positive regulation of nitrogen compound metabolic process	18/30	306/751	0.02361	0.379637	0.357786	APLNR/IL34/BAX/BRD4/BBC3/CASP3/ARID1A/HRAS/WNT5A/CCNB1/IFI16/VEGFB/LGALS9/CSF2/ENO1/IKBKG/TRIM21/FADD
GO:0043066	negative regulation of apoptotic process	11/30	151/751	0.024053	0.382166	0.360169	NQO1/GCLM/BAX/GCLC/CASP3/HRAS/WNT5A/ARG2/CSF2/ENO1/FADD
GO:0009416	response to light stimulus	4/30	29/751	0.024474	0.382166	0.360169	SLC7A11/BAX/DDB2/CASP3
GO:0032757	positive regulation of interleukin-8 production	4/30	29/751	0.024474	0.382166	0.360169	CD2/WNT5A/LGALS9/FADD
GO:0097190	apoptotic signaling pathway	9/30	113/751	0.025624	0.385122	0.362955	GCLM/BAX/BBC3/CASP3/HRAS/IFI16/CSF2/ENO1/FADD
GO:0045787	positive regulation of cell cycle	5/30	44/751	0.025979	0.385122	0.362955	BAX/BRD4/WNT5A/CCNB1/TRIM21
GO:0043069	negative regulation of programmed cell death	11/30	153/751	0.026434	0.385122	0.362955	NQO1/GCLM/BAX/GCLC/CASP3/HRAS/WNT5A/ARG2/CSF2/ENO1/FADD
GO:0010389	regulation of G2/M transition of mitotic cell cycle	3/30	17/751	0.026797	0.385122	0.362955	BRD4/CCNB1/PSMB5
GO:0016331	morphogenesis of embryonic epithelium	3/30	17/751	0.026797	0.385122	0.362955	CASP3/WNT5A/WNT2B
GO:0043200	response to amino acid	3/30	17/751	0.026797	0.385122	0.362955	NQO1/GCLM/CASP3
GO:0045824	negative regulation of innate immune response	3/30	17/751	0.026797	0.385122	0.362955	IFI16/LGALS9/TRIM21

GO:0046660	female sex differentiation	3/30	17/751	0.026797	0.385122	0.362955	BAX/CASP3/WNT5A
GO:0050798	activated T cell proliferation	3/30	17/751	0.026797	0.385122	0.362955	CASP3/LGALS9/FADD
GO:0002218	activation of innate immune response	4/30	30/751	0.027457	0.387743	0.365426	HRAS/IFI16/PSMB5/IKBKG
GO:0043280	positive regulation of cysteine-type endopeptidase activity involved in apoptotic process	4/30	30/751	0.027457	0.387743	0.365426	BAX/BBC3/LGALS9/FADD
GO:0009894	regulation of catabolic process	7/30	78/751	0.028169	0.390929	0.368428	BAX/CASP3/WNT5A/IFI16/PSMB5/IKBKG/TRIM21
GO:0007249	I-kappaB kinase/NF-kappaB signaling	6/30	61/751	0.028346	0.390929	0.368428	BRD4/WNT5A/LGALS9/IKBKG/TRIM21/FADD
GO:2000116	regulation of cysteine-type endopeptidase activity	5/30	45/751	0.028405	0.390929	0.368428	BAX/BBC3/IFI16/LGALS9/FADD
GO:1901576	organic substance biosynthetic process	19/30	336/751	0.028733	0.392116	0.369547	GCLM/SLC7A11/BAX/BRD4/GCLC/ARID1A/HRAS/WNT5A/ARG2/CCNB1/IFI16/VEGFB/LGALS9/CSF2/ENO1/PSMB5/IKBKG/TRIM21/FADD
GO:0032677	regulation of interleukin-8 production	4/30	31/751	0.030653	0.412299	0.388568	CD2/WNT5A/LGALS9/FADD
GO:0001655	urogenital system development	5/30	46/751	0.030973	0.412299	0.388568	BAX/WNT5A/ARG2/WNT2B/FADD
GO:0048872	homeostasis of number of cells	5/30	46/751	0.030973	0.412299	0.388568	SLC7A11/BAX/CASP3/LGALS9/FADD
GO:1902749	regulation of cell cycle G2/M phase transition	3/30	18/751	0.031297	0.413227	0.389443	BRD4/CCNB1/PSMB5
GO:0002687	positive regulation of leukocyte migration	5/30	47/751	0.033686	0.425523	0.401031	IL34/WNT5A/VEGFB/LGALS9/FADD
GO:0043603	cellular amide metabolic process	5/30	47/751	0.033686	0.425523	0.401031	GCLM/SLC7A11/GCLC/CASP3/ARG2
GO:0090304	nucleic acid metabolic process	17/30	292/751	0.033696	0.425523	0.401031	BAX/BRD4/GCLC/DDB2/CASP3/ARID1A/HRAS/WNT5A/CCNB1/IFI16/LGALS9/CSF2/ENO1/PSMB5/IKBKG/TRIM21/FADD
GO:0032637	interleukin-8 production	4/30	32/751	0.034063	0.425523	0.401031	CD2/WNT5A/LGALS9/FADD
GO:0032651	regulation of interleukin-1 beta production	4/30	32/751	0.034063	0.425523	0.401031	WNT5A/ARG2/IFI16/LGALS9

GO:0071675	regulation of mononuclear cell migration	4/30	32/751	0.034063	0.425523	0.401031	IL34/WNT5A/LGALS9/FADD
GO:0090068	positive regulation of cell cycle process	4/30	32/751	0.034063	0.425523	0.401031	BAX/BRD4/WNT5A/CCNB1
GO:0061024	membrane organization	6/30	64/751	0.035118	0.431955	0.407092	SLC7A11/BAX/BBC3/CD2/WNT5A/CCNB1
GO:0006725	cellular aromatic compound metabolic process	18/30	318/751	0.03597	0.431955	0.407092	SLC7A11/BAX/BRD4/GCLC/DDB2/CASP3/ARID1A/HRAS/WNT5A/CCNB1/IFI16/LGALS9/CSF2/ENO1/PSMB5/IKBKG/TRIM21/FADD
GO:0031325	positive regulation of cellular metabolic process	18/30	318/751	0.03597	0.431955	0.407092	APLNR/IL34/BAX/BRD4/BBC3/CASP3/ARID1A/HRAS/WNT5A/CCNB1/IFI16/VEGFB/LGALS9/CSF2/ENO1/IKBKG/TRIM21/FADD
GO:0000086	G2/M transition of mitotic cell cycle	3/30	19/751	0.036174	0.431955	0.407092	BRD4/CCNB1/PSMB5
GO:0001101	response to acid chemical	3/30	19/751	0.036174	0.431955	0.407092	NQO1/GCLM/CASP3
GO:0090150	establishment of protein localization to membrane	3/30	19/751	0.036174	0.431955	0.407092	BAX/BBC3/HRAS
GO:0001654	eye development	4/30	33/751	0.03769	0.440345	0.415	SLC7A11/BAX/WNT5A/WNT2B
GO:0002690	positive regulation of leukocyte chemotaxis	4/30	33/751	0.03769	0.440345	0.415	IL34/WNT5A/VEGFB/LGALS9
GO:0007548	sex differentiation	4/30	33/751	0.03769	0.440345	0.415	BAX/CASP3/WNT5A/WNT2B
GO:2001234	negative regulation of apoptotic signaling pathway	5/30	49/751	0.039556	0.440737	0.415369	GCLM/BAX/CSF2/ENO1/FADD
GO:0031329	regulation of cellular catabolic process	6/30	66/751	0.040185	0.440737	0.415369	BAX/CASP3/IFI16/PSMB5/IKBKG/TRIM21
GO:0044260	cellular macromolecule metabolic process	23/30	451/751	0.040953	0.440737	0.415369	APLNR/IL34/PARP12/BAX/BRD4/BBC3/GCLC/DDB2/CASP3/ARID1A/HRAS/WNT5A/CCNB1/IFI16/VEGFB/LGALS9/CSF2/ENO1/PSMB5/IKBKG/TRIM21/FADD/GAS1
GO:0000209	protein polyubiquitination	3/30	20/751	0.041422	0.440737	0.415369	DDB2/PSMB5/TRIM21
GO:0006919	activation of cysteine-type endopeptidase activity involved in apoptotic process	3/30	20/751	0.041422	0.440737	0.415369	BAX/BBC3/FADD
GO:0008637	apoptotic mitochondrial changes	3/30	20/751	0.041422	0.440737	0.415369	GCLM/BAX/BBC3

GO:0044839	cell cycle G2/M phase transition	3/30	20/751	0.041422	0.440737	0.415369	BRD4/CCNB1/PSMB5
GO:0048145	regulation of fibroblast proliferation	3/30	20/751	0.041422	0.440737	0.415369	BAX/WNT5A/CCNB1
GO:0048592	eye morphogenesis	3/30	20/751	0.041422	0.440737	0.415369	BAX/WNT5A/WNT2B
GO:0071248	cellular response to metal ion	3/30	20/751	0.041422	0.440737	0.415369	NQO1/WNT5A/CCNB1
GO:0032611	interleukin-1 beta production	4/30	34/751	0.041535	0.440737	0.415369	WNT5A/ARG2/IFI16/LGALS9
GO:0043604	amide biosynthetic process	4/30	34/751	0.041535	0.440737	0.415369	GCLM/SLC7A11/GCLC/ARG2
GO:1901360	organic cyclic compound metabolic process	18/30	323/751	0.042445	0.440737	0.415369	SLC7A11/BAX/BRD4/GCLC/DDB2/CASP3/ARID1A/HRAS/WNT5A/CCNB1/IFI16/LGALS9/CSF2/ENO1/PSMB5/IKBKG/TRIM21/FADD
GO:0032649	regulation of interferon-gamma production	5/30	50/751	0.042716	0.440737	0.415369	CD2/HRAS/WNT5A/LGALS9/FADD
GO:2001233	regulation of apoptotic signaling pathway	6/30	67/751	0.042888	0.440737	0.415369	GCLM/BAX/BBC3/CSF2/ENO1/FADD
GO:0019438	aromatic compound biosynthetic process	16/30	276/751	0.043799	0.440737	0.415369	SLC7A11/BAX/BRD4/GCLC/ARID1A/HRAS/WNT5A/CCNB1/IFI16/LGALS9/CSF2/ENO1/PSMB5/IKBKG/TRIM21/FADD
GO:0031347	regulation of defense response	10/30	144/751	0.044192	0.440737	0.415369	BRD4/HRAS/WNT5A/ARG2/IFI16/LGALS9/PSMB5/IKBKG/TRIM21/FADD
GO:0007049	cell cycle	9/30	124/751	0.044556	0.440737	0.415369	BAX/BRD4/CASP3/HRAS/WNT5A/CCNB1/PSMB5/TRIM21/GAS1
GO:0048880	sensory system development	4/30	35/751	0.045599	0.440737	0.415369	SLC7A11/BAX/WNT5A/WNT2B
GO:0150063	visual system development	4/30	35/751	0.045599	0.440737	0.415369	SLC7A11/BAX/WNT5A/WNT2B
GO:0035556	intracellular signal transduction	17/30	301/751	0.045642	0.440737	0.415369	APLNR/IL34/BAX/BRD4/BBC3/CASP3/HRAS/WNT5A/CCNB1/IFI16/LGALS9/CSF2/ENO1/PSMB5/IKBKG/TRIM21/FADD
GO:0006351	transcription, DNA-templated	15/30	254/751	0.04575	0.440737	0.415369	BAX/BRD4/GCLC/ARID1A/HRAS/WNT5A/CCNB1/IFI16/LGALS9/CSF2/ENO1/PSMB5/IKBKG/TRIM21/FADD
GO:0097659	nucleic acid-templated transcription	15/30	254/751	0.04575	0.440737	0.415369	BAX/BRD4/GCLC/ARID1A/HRAS/WNT5A/CCNB1/IFI16/LGALS9/CSF2/ENO1/PSMB5/IKBKG/

							TRIM21/FADD
GO:0032609	interferon-gamma production	5/30	51/751	0.046028	0.440737	0.415369	CD2/HRAS/WNT5A/LGALS9/FADD
GO:0009056	catabolic process	10/30	145/751	0.046141	0.440737	0.415369	BAX/CASP3/WNT5A/ARG2/CCNB1/IFI16/ENO1/PSMB5/IKBKG/TRIM21
GO:0009411	response to UV	3/30	21/751	0.047039	0.440737	0.415369	BAX/DDB2/CASP3
GO:0030225	macrophage differentiation	3/30	21/751	0.047039	0.440737	0.415369	IL34/CSF2/FADD
GO:0048144	fibroblast proliferation	3/30	21/751	0.047039	0.440737	0.415369	BAX/WNT5A/CCNB1
GO:0071677	positive regulation of mononuclear cell migration	3/30	21/751	0.047039	0.440737	0.415369	WNT5A/LGALS9/FADD
GO:0032774	RNA biosynthetic process	15/30	255/751	0.047339	0.440737	0.415369	BAX/BRD4/GCLC/ARID1A/HRAS/WNT5A/CCNB1/IFI16/LGALS9/CSF2/ENO1/PSMB5/IKBKG/TRIM21/FADD
GO:0080134	regulation of response to stress	13/30	210/751	0.047765	0.440737	0.415369	SLC7A11/BAX/BRD4/HRAS/WNT5A/ARG2/IFI16/LGALS9/ENO1/PSMB5/IKBKG/TRIM21/FADD

Table S3. Multivariate Regression Analysis for Change in SPMA.

Variable	RR[a]	2.5%	97.5%	*p*-Value
CSF2RB	1.050	0.774	1.424	0.76
CXCL6	1.086	0.924	1.277	0.33
TAPBP	1.145	0.528	2.483	0.73
CXCL8	1.170	0.919	1.490	0.21
HCK	1.400	0.947	2.071	0.1
GPC4	1.323	1.048	1.671	0.027
CD69	1.294	0.984	1.701	0.077
IFITM1	0.599	0.403	0.891	0.018
ITGAX	0.836	0.567	1.232	0.37
CD44	1.023	0.731	1.432	0.89
LAMC2	1.583	1.258	1.991	< 0.001
IL6R	1.217	0.858	1.725	0.28
ICAM3	1.033	0.671	1.590	0.89
SAMSN1	0.981	0.678	1.421	0.92
IFITM2	0.880	0.557	1.390	0.59
HLA.C	0.770	0.389	1.525	0.46
FCGRT	0.951	0.621	1.455	0.82
CXCL1	0.899	0.649	1.244	0.53
HLA.B	0.916	0.534	1.572	0.75
ITGB2	1.348	0.949	1.913	0.11
SGK1	1.048	0.786	1.398	0.75
